# Severe acute tumor lysis syndrome in patients with germ-cell tumors

**DOI:** 10.4103/0970-1591.44267

**Published:** 2008

**Authors:** Guilherme Alvarenga Feres, Jorge Ibrain Figueira Salluh, Carlos Gil Ferreira, Marcio Soares

**Affiliations:** Intensive Care Unit, Instituto Nacional de Câncer (INCA), Rio de Janeiro, RJ, Brazil; 1 Department of Clinical Research, Instituto Nacional de Câncer (INCA), Rio de Janeiro, RJ, Brazil

**Keywords:** Acute renal failure, germ-cell tumors, tumor lysis syndrome

## Abstract

Germ-cell tumors are a high-proliferative type of cancer that may evolve to significant bulky disease. Tumor lysis syndrome is rarely reported in this setting. The reports of three patients with germ-cell tumors who developed severe acute tumor lysis syndrome following the start of their anticancer therapy are presented. All patients developed renal dysfunction and multiorgan failure. Patients with extensive germ-cell tumors should be kept on close clinical and laboratory monitoring. Physicians should be aware of this uncommon but severe complication and consider early admission to the intensive care unit for the institution of measures to prevent acute renal failure.

## INTRODUCTION

Acute tumor lysis syndrome (ATLS) is a clinical condition that results from the massive destruction and lysis of malignant cells, and subsequent release of intracellular ions and metabolites into the bloodstream leading to hyperkalemia, hyperphosphatemia, hyperuricemia, and hypocalcemia. ATLS is accompanied by renal failure and metabolic acidosis thus increasing the risk of death. The syndrome is usually a consequence of the treatment of high-grade lymphoproliferative malignancies. It is rarely observed in patients with solid tumors and even less frequent is its spontaneous presentation.[1–3] Germ-cell tumors are high-proliferative malignancies that can become bulky in disseminated disease. Nevertheless, ATLS has been rarely reported in this setting.[3–5] This manuscript presents three cases of patients with germ-cell tumors who were admitted to our intensive care unit (ICU) with severe ATLS following the start of anticancer therapy.

## CASE REPORTS

### Patient 1

A 41-year-old male presented with left testicular mass, weight loss, dry cough followed by hemoptysis and progressive dyspnea. Computed tomography (CT) scans showed a large retroperitoneal mass and metastases to the lungs, liver, and spleen. There were high levels of human chorionic gonadotropin (HCG) (317.718 mU/ml) and of α-fetoprotein (93 ng/ml). The patient underwent a radical left orchiectomy and the hystopathological examination showed a mixed germ-cell tumor (embryonal carcinoma, 65%; seminoma, 35%). On the first day of chemotherapy with bleomycin, etoposide, and cisplatin, he developed massive hemoptisys, acute respiratory failure, and cardiopulmonary arrest. Cardiopulmonary resuscitation was successfully performed. On day 2, he evolved with metabolic acidosis, hyperphosphatemia (8.8 mg/dl), hyperuricemia (9.4 mg/dl), hypocalcemia (6.1 mg/dl), and hyperkalemia (6.7 mEq/l). Chemotherapy was interrupted and hemodyalisis was started. However, the patient died on day 7 due to multiple organ failure.

### Patient 2

**Figure 1:**
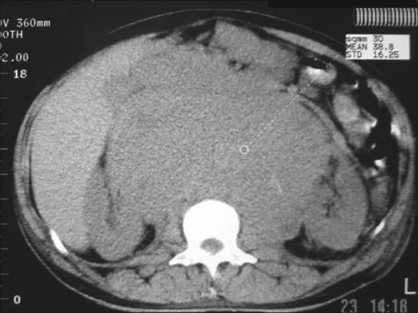
Patient 2: Abdominal computed tomography (CT) revealing large retroperitoneal mass

**Figure 2:**
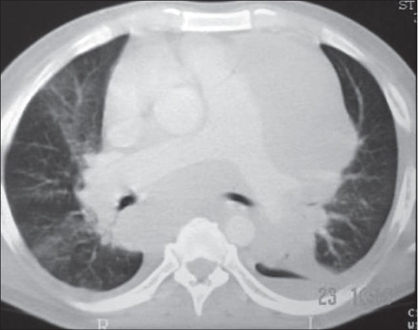
Patient 2: Chest computed tomography (CT) showing large mediastinal mass involving aortic arch and compressing main bronchi

A 39-year-old male presented with weight loss, progressive dyspnea, and right testicular enlargement. CT scan showed large mediastinal, intraperitoneal, and retroperitoneal masses [Figure 1, 2]. Serum HCG level was 202 mUI/ml, lactate dehydrogenase (LDH) of 13.666 U/l, and the histopathological analysis of the testis revealed a seminomatous tumor. He received chemotherapy with etoposide and carboplatin. Subsequently, the patient developed respiratory failure and metabolic acidosis. Alopurinol, urine alkalinization, and vigorous hydration were initiated concerning the risk of ATLS. On day 3, he evolved with hyperkalemia (6.0 mEq/dl), hyperphosphatemia (8.3 mg/dl), hyperuricemia (22 mg/dl), renal failure and shock, and required hemodyalisis. On day 6, abdominal and mediastinal masses diminished and overall patient′s clinical condition improved including progressive recovery of renal function. The patient was extubated on day 12 and discharged from the ICU on day 15. Before ICU discharge, a significant reduction in tumor markers was observed (HCG 3 mU/ml and LDH 4743 U/l). In the wards, he received another cycle of chemotherapy with bleomycin in addition to etoposide and carboplatin. On day 30 of hospital admission, he was discharged home. Sixteen months following hospital discharge, the patient had no evidence of disease.

### Patient 3

A 30-year-old male presented with back pain, bilateral leg edema, and dyspnea because of a large retroperitoneal mass and multiple pulmonary nodules. Ultrasound examination disclosed hydronephrosis of the right kidney and deep vein thrombosis. There was no palpable testicular mass on physical examination, but a right testicular nodule was disclosed by ultrasound examination. Tumor markers showed a serum α-fetoprotein of 19.078 U/l, HCG of 5.4 U/l, and LDH of 7.150 U/l. The patient underwent right radical orchiectomy and nonseminomatous tumor was diagnosed. Etoposide and carboplatin were started. However, on day 1 of chemotherapy, he developed acute respiratory failure requiring mechanical ventilation and was transferred to the ICU. Vigorous hydration and alopurinol were started concerning the risk of tumor lysis syndrome. Nonetheless, the patient developed low urine output, metabolic acidosis, azotemia, hyperphosphatemia, hyperuricemia, hyperkalemia, and hemodynamic instability. Chemotherapy was stopped and broad-spectrum antibiotics and hemodyalisis were started. On day 6, he presented with progressive pancytopenia requiring the use of filgrastim. The patient evolved with progressive improvement of his clinical condition, mechanical ventilation was discontinued on day 22, and he was discharged to the wards on day 24. In the wards, his renal function improved allowing to the discontinuation of hemodyalisis. The patient went home on day 42 with normal renal function and lower tumor markers levels (α-fetoprotein of 1.982 U/l, HCG of 4.5 U/l, and LDH of 1.379 U/l.). He received three additional courses of bleomycin, etoposide, and carboplatin with no serious side effects. Two months later, he underwent an exploratory laparotomy and there was no evidence of retroperitoneal masses. However, there was disease progression characterized by the development of recurrent metastases in the central nervous system refractory to surgery, chemotherapy, and radiation therapy. The patient died 20 months after index hospital admission.

## DISCUSSION

The reports of three patients with extensive disseminated germ-cell tumors who developed severe ATLS following the start of chemotherapy were presented. ATLS has been occasionally described in patients with solid tumors such as melanoma, medulloblastoma, small-cell lung cancer, neuroblastoma, and germ-cell tumors.[1–5] The observation of ATLS in Patient 1 raised concern over the possibility of the development of this syndrome in Patients 2 and 3, allowing us to implement strict clinical and laboratory monitoring, and the institution of prophylatic measures such as vigorous hydration and use of alopurinol. Although, Patients 2 and 3 had also evolved with acute renal failure, they were successfully released home in contrast to Patient 1. All of our three patients became severely ill with acute renal failure, acute respiratory failure, and shock. Kirch and colleagues[4] reported 16 patients with germ-cell tumors with diffuse pulmonary metastases who developed acute respiratory failure following the start of chemotherapy. Out of them, nine required mechanical ventilation and all but one patient died. The use of bleomycin is a controversial issue in patients with germ-cell tumor and pulmonary involvement, but in this scenario the development of respiratory failure may not be related to bleomycin use.[4] Physicians should be aware of this uncommon but severe complication in patients with extensive disseminated germ-cell tumors. They should keep their patients on close clinical and laboratory monitoring and consider early transfer to the ICU and institution of measures to prevent acute renal failure.
